# Influential Parameters for the Analysis of Intracellular Parasite Metabolomics

**DOI:** 10.1128/mSphere.00097-18

**Published:** 2018-04-18

**Authors:** Maureen A. Carey, Vincent Covelli, Audrey Brown, Gregory L. Medlock, Mareike Haaren, Jessica G. Cooper, Jason A. Papin, Jennifer L. Guler

**Affiliations:** a Department of Microbiology, Immunology, and Cancer Biology, University of Virginia School of Medicine, Charlottesville, Virginia, USA; b Division of Infectious Disease and International Health, University of Virginia School of Medicine, Charlottesville, Virginia, USA; c Department of Biology, University of Virginia, Charlottesville, Virginia, USA; d Department of Biomedical Engineering, University of Virginia, Charlottesville, Virginia, USA; University at Buffalo

**Keywords:** *Plasmodium falciparum*, apicomplexan parasites, intracellular pathogen, metabolomics

## Abstract

Metabolomics is increasingly popular for the study of pathogens. For the malaria parasite Plasmodium falciparum, both targeted and untargeted metabolomics have improved our understanding of pathogenesis, host-parasite interactions, and antimalarial drug treatment and resistance. However, purification and analysis procedures for performing metabolomics on intracellular pathogens have not been explored. Here, we purified *in vitro*-grown ring-stage intraerythrocytic P. falciparum parasites for untargeted metabolomics studies; the small size of this developmental stage amplifies the challenges associated with metabolomics studies as the ratio between host and parasite biomass is maximized. Following metabolite identification and data preprocessing, we explored multiple confounding factors that influence data interpretation, including host contamination and normalization approaches (including double-stranded DNA, total protein, and parasite numbers). We conclude that normalization parameters have large effects on differential abundance analysis and recommend the thoughtful selection of these parameters. However, normalization does not remove the contribution from the parasite’s extracellular environment (culture media and host erythrocyte). In fact, we found that extraparasite material is as influential on the metabolome as treatment with a potent antimalarial drug with known metabolic effects (artemisinin). Because of this influence, we could not detect significant changes associated with drug treatment. Instead, we identified metabolites predictive of host and medium contamination that could be used to assess sample purification. Our analysis provides the first quantitative exploration of the effects of these factors on metabolomics data analysis; these findings provide a basis for development of improved experimental and analytical methods for future metabolomics studies of intracellular organisms.

**IMPORTANCE** Molecular characterization of pathogens such as the malaria parasite can lead to improved biological understanding and novel treatment strategies. However, the distinctive biology of the *Plasmodium* parasite, including its repetitive genome and the requirement for growth within a host cell, hinders progress toward these goals. Untargeted metabolomics is a promising approach to learn about pathogen biology. By measuring many small molecules in the parasite at once, we gain a better understanding of important pathways that contribute to the parasite’s response to perturbations such as drug treatment. Although increasingly popular, approaches for intracellular parasite metabolomics and subsequent analysis are not well explored. The findings presented in this report emphasize the critical need for improvements in these areas to limit misinterpretation due to host metabolites and to standardize biological interpretation. Such improvements will aid both basic biological investigations and clinical efforts to understand important pathogens.

## Abstract (precis)

Molecular characterization of pathogens such as the malaria parasite can lead to improved biological understanding and novel treatment strategies. However, the distinctive biology of the *Plasmodium* parasite, including its repetitive genome and the requirement for growth within a host cell, hinders progress toward these goals. Untargeted metabolomics is a promising approach to learn about pathogen biology. By measuring many small molecules in the parasite at once, we gain a better understanding of important pathways that contribute to the parasite’s response to perturbations such as drug treatment. Although increasingly popular, approaches for intracellular parasite metabolomics and subsequent analysis are not well explored. The findings presented in this report emphasize the critical need for improvements in these areas to limit misinterpretation due to host metabolites and to standardize biological interpretation. Such improvements will aid both basic biological investigations and clinical efforts to understand important pathogens.

## INTRODUCTION

Malaria continues to be responsible for hundreds of thousands of deaths annually, most of which result from infection with the protozoan parasite Plasmodium falciparum (1). Characterization of the biology of this important pathogen can lead to improved treatment strategies. Omics approaches, such as genomics, transcriptomics, and proteomics, are widely used, but the limited annotation of the parasite’s genome makes these data sets challenging to interpret. One way to alleviate this lack of functional knowledge is to use network-based modeling to contextualize noisy or sparse data and facilitate the interpretation of complex data (2). Additionally, the measurement of direct mediators of the phenotype, such as signaling and biosynthetic metabolites, can improve the ability to characterize phenotypes mediated by proteins that are not yet annotated in the genome. For this reason, metabolomics is becoming increasingly popular in studies of intraerythrocytic stages of P. falciparum (3–12). These studies have improved our understanding of malaria pathogenesis (7), strain-specific phenotypes (11), and host-parasite interactions (9). Recent studies have successfully identified metabolic signatures that correlate well with biological function, such as time- and dose-dependent responses to antimalarial treatment (3, 5) and resistance-conferring mutations (12).

Previous studies on P. falciparum have been confined to the larger, late-intra-erythrocyte-stage parasites. This is mainly due to the characteristics of the available purification approaches used; for example, magnetic purification specifically enriches late-stage parasites that contain paramagnetic hemozoin while excluding early ring stages and uninfected host cells (13). Accordingly, the study of the smaller, early-ring-stage parasite is more challenging due to an inability to isolate adequate amounts of parasite material from host material (12). However, specific functionality (i.e., artemisinin resistance) can be observed only in the early parasite stages and metabolic details would greatly advance our understanding of such phenotypes.

There are distinct challenges that need to be considered in performing metabolomic studies in obligate intracellular pathogens such as P. falciparum; chief among these are acquiring adequate material and the potential for contamination from host cells. Due to inefficient purification methods, samples typically have few parasites and yet abundant host erythrocyte material. Uninfected host cells are often >10 times more prevalent than P. falciparum-infected host cells in laboratory culture and clinical infections, and the host erythrocyte contains up to 10-fold more cellular material (14, 15).

In this study, we sought to define critical parameters that can be used to overcome these challenges and facilitate the collection of high-quality metabolomics data. We chose to investigate an extreme case, namely, metabolically perturbing early-ring-stage P. falciparum parasites, to determine if the extensive extraparasite contamination present after employment of commonly used isolation methods can be removed analytically. We show that both the choice of analytic parameters (in particular, the normalization approach) and extraparasite contamination heavily influence the interpretation of metabolic changes. However, even appropriate normalization fails to remove environmental noise completely. Contamination from the media and host cells is as influential on the metabolome as sample treatment. Thus, we propose that the combination of improved purification and improved analytic parameters could generate more accurate measures of the metabolome, increasing the utility of untargeted metabolomics to investigate intracellular parasite biology.

## RESULTS

### Metabolomics.

**FIG 1:**
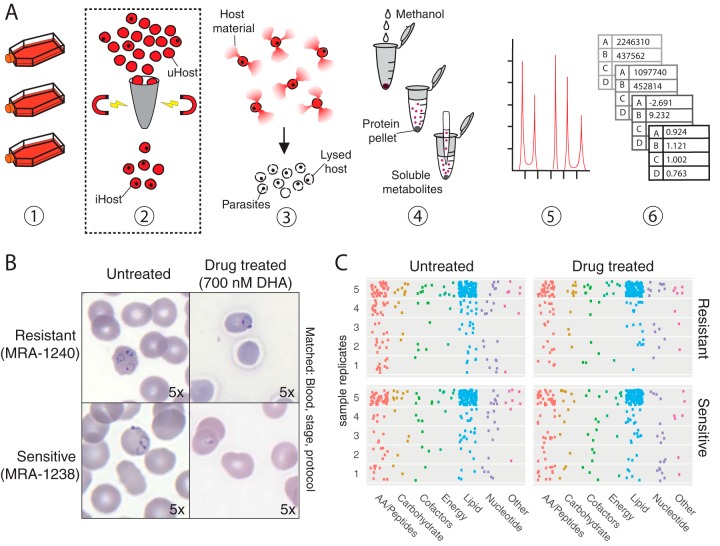
Metabolomics pipeline and metabolite identification. (A) Metabolomics purification and analysis pipeline. (Step 1) Laboratory-adapted P. falciparum clones are cultured in host erythrocytes. Parasite count is collected at this step (total erythrocyte number multiplied by percent parasitemia yields total parasite value; see Materials and Methods). (Step 2) If enriching for late-stage parasites is desired, cultures are passed through a magnetic column to retain paramagnetic late-stage-infected erythrocytes. Note that this was not done for the present study. iHost, infected host erythrocytes; uHost, uninfected host erythrocytes. (Step 3) Host erythrocytes are lysed using saponin, but parasites remain intact. Samples are washed to remove hemoglobin and other intracellular host material and quenched on liquid nitrogen. Total protein is quantified at this step (prior to freezing). (Step 4) Soluble metabolites are extracted from precipitated protein using methanol and centrifugation. Double-stranded DNA is quantified at this step. (Step 5) Metabolites are separated via liquid chromatography and identified using mass spectroscopy. Metabolite spectra are compared to a library of authenticated standard metabolites for high-confidence identification. (Step 6) Abundance data for each metabolite are normalized to an appropriate parameter (i.e., DNA content or parasite number), log transformed, centered with respect to the median, and scaled with respect to variances, prior to employing statistical comparisons. (B) Experimental comparison. All samples were grown in RPMI media supplemented with AlbuMAX and hypoxanthine and with one of three blood batches (matched across treatment conditions). At the early ring stage (<3 h postinvasion), 10 samples were treated with dihydroartemisinin (DHA; 700 nM) for 6 h and 10 samples were matched with respect to protocol and condition (blood batch, medium batch, and stage) without drug treatment (see Table S3). Images shown were taken at the 6-h time point (×100 magnification); dormancy was observed at 24 h. (C) Summary of identified metabolites. Metabolites (each represented by one point) from various metabolic subgroups were not uniformly detected in all five replicates for any sample group. How frequently a metabolite was measured across replicates is indicated by the metabolite point placed in data corresponding to 1 to 5 replicates (*y* axis). The majority of metabolites detected were lipid species, as indicated by the large number of blue dots. A full list of identified metabolites is provided in the supplemental material.

We conducted metabolomics on early-ring-stage (0 to 3 h) Plasmodium falciparum parasites lysed from host erythrocytes. Two parasite clones were grown in matched conditions, lysed and washed from the host cell, and analyzed via ultra-high-performance liquid chromatography coupled with mass spectrometry (UPLC/MS) (Fig. 1A). Prior to isolation, each clone (representing either a drug-sensitive or a drug-resistant line) was either left untreated or treated with 700 nM dihydroartemisinin (for 6 h), generating four sample groups with matched blood batches, media, and purification approaches (Fig. 1B). Dihydroartemisinin, the active component of the antimalarial artemisinin, is a known metabolic disruptor (3, 5, 16). Both sensitive and resistant parasites are known to enter a unique metabolic state, called dormancy, following treatment. Dormancy is characterized by reduced metabolic activity (17–19); thus, treated ring-stage parasites should have a metabolome distinct from that seen with untreated parasites.

Mass spectrometry analysis of these samples detected 297 identifiable metabolites; 155 metabolites were detected in every sample. Samples contained between 182 and 267 metabolites. The detected metabolites represented 10 energy-associated metabolites, 159 lipid species, 108 peptides and amino acids, 40 nucleotides, 28 cofactors, 20 carbohydrates, and 10 in other categories (see Table S1 in the supplemental material). Lipid species were the most consistently detected metabolites in every sample (as measured by the percentage of metabolite found in every sample), and amino acids were often unique to individual samples (Fig. 1C). Several metabolites were measured that are not known to be part of P. falciparum metabolism, including kynurenine (detected in 25% of samples), phenol red (phenolsulfonphthalein; detected in 95% of samples), and HEPES (detected in all samples; see Table S1).

10.1128/mSphere.00097-18.2 TABLE S1  Metabolomics data. Metabolite abundance data were provided by Metabolon, Inc., prior to data processing and analysis. See GitHub (specifically, https://github.com/gulermalaria/metabolomics/blob/master/met_methods_raw_data.txt) or the Excel spreadsheet. Download TABLE S1, XLSX file, 0.1 MB. Copyright © 2018 Carey et al. 2018 Carey et al. This content is distributed under the terms of the Creative Commons Attribution 4.0 International license.

### Host contamination.

**FIG 2:**
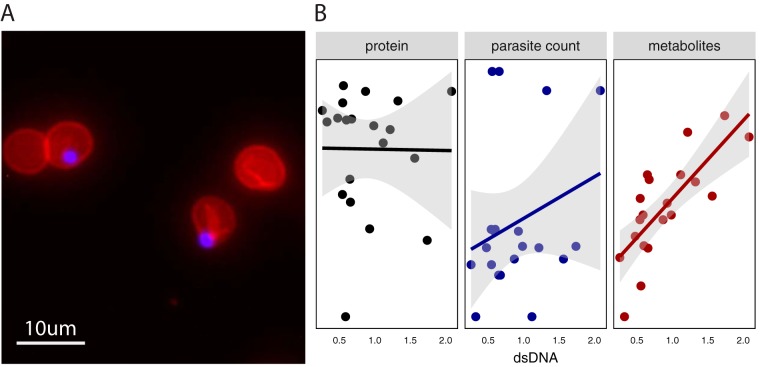
Host persistence is detected using multiple approaches. (A) Visualization of parasites within erythrocyte ghosts. Fluorescent imaging (×40 magnification) reveals parasites (blue, DAPI) retained within erythrocyte ghosts (red, phycoerythrin-conjugated CD235a antibody) following saponin treatment. Approximately 70% of the parasites remain associated with host membranes (see Table S2). (B) Sample characteristics. Samples were evaluated for levels of double-stranded DNA (dsDNA; quantified in micrograms per milliliter on the *x* axis), protein amounts (black; quantified in micrograms on the *y* axis [ranging from 67.0641 to 130.0936 μg] in the left panel), and parasite counts (blue; quantified on the *y* axis [ranging from 1,306,500 to 6,946,875 parasites] in the center panel) prior to analysis. The total number of metabolites detected per sample (red; quantified on the *y* axis [ranging from 182 to 267 metabolites] in the right panel) was significantly correlated with sample dsDNA quantification (*P* = 9.8 × 10^−5^; *r*^2^ = 0.76). Protein amount and parasite count were not significantly correlated with dsDNA. The fit line uses a linear model, and the shaded region represents the standard error.

Despite implementation of the current best practices, including erythrocyte lysis and washing steps to remove parasites from their intracellular milieu (Fig. 1A; see, e.g., references 3 and 8), parasite separation from the host is poor. Microscopy confirmed that the parasites lysed from host cells remained embedded in erythrocyte membranes and that washes failed to isolate parasite material (Fig. 2A) (20). Importantly, over 68% of parasites remained associated with the host membrane (Table S2). This result emphasized that erythrocyte “ghosts” (cell membranes with associated metabolites) remained abundant in the sample and could have heavily contributed to the metabolome. Thus, we sought analytic approaches to remove host contamination *post hoc*.

10.1128/mSphere.00097-18.3 TABLE S2  Host entrapment of purified parasites. Parasites remain in host cells following purification. Laboratory-adapted P. falciparum clones (BEI Resources, NIAID, NIH; Plasmodium falciparum patient line strain E/MRA-1000 or strain IPC 5202/MRA-1238, contributed by Didier Ménard) with >60% rings were lysed using 0.15% saponin as described previously and in Materials and Methods. Samples were washed twice using 1× PBS (Sigma-Aldrich, St. Louis, MO) and centrifugation at 2,000 × *g* for 5 min. Samples were then stained on slides with either DAPI (Sigma-Aldrich, St. Louis, MO) at 1:20,000 or CD235a-PE antibody (Thermo Fisher Scientific, Waltham, MA) at 1:100 for fluorescence microscopy. Fluorescent images were acquired using an Evos FL cell imaging system (Thermo Fisher Scientific, Waltham, MA). Parasites not associated with erythrocyte membranes were counted. These samples were protocol matched to metabolomics samples but were grown from separate cultures. Download TABLE S2, DOCX file, 0.01 MB. Copyright © 2018 Carey et al. 2018 Carey et al. This content is distributed under the terms of the Creative Commons Attribution 4.0 International license.

### Normalization.

We first explored the use of normalization with three distinct approaches. Metabolomics preprocessing methods can influence results (21, 22), but the role of normalization, particularly in intracellular pathogens, has not been extensively explored. Both host- and parasite-derived metrics (double-stranded DNA or dsDNA, protein, and parasite levels) were evaluated in the experimental setup (Fig. 1A). Sample replicates contained 1.3 to 6.9 million parasites (Table S3). As expected, no two normalization metrics were correlated across samples (Fig. 2B; see the supplemental materials for codes). Metabolite yield (as measured by the number of identified metabolites) was correlated only with DNA abundance (*P* = 9.8 × 10^−5^, *r*^2^ = 0.76) (Fig. 2B), indicating that DNA abundance is associated best with total biomass.

10.1128/mSphere.00097-18.4 TABLE S3  Parasite sample reference table. Parasite samples were quantified by protein, DNA, parasite number, parasitemia, and stage distribution data. Download TABLE S3, DOCX file, 0.01 MB. Copyright © 2018 Carey et al. 2018 Carey et al. This content is distributed under the terms of the Creative Commons Attribution 4.0 International license.

Initially, we anticipated that dsDNA should come primarily from the parasite fraction, as host erythrocytes are anucleate and growth medium does not contain any intact DNA; however, we found that host cells and AlbuMAX (a medium component) did contribute to sample dsDNA (see Fig. S1 in the supplemental material). Protein was likely also derived from all three culture components, namely, parasite, host erythrocyte, and media (via AlbuMAX supplementation). Although parasite counts represent a direct measure of the parasite fraction, this variable was collected several steps upstream of metabolome quantification (Fig. 1A) and may have been suboptimal compared to metrics collected later in the pipeline.

10.1128/mSphere.00097-18.1 FIG S1  DNA contribution from host erythrocyte and media. Erythrocytes contribute DNA despite being anucleated. Measurement of host-derived dsDNA levels was performed by incubating uninfected erythrocytes at 3% hematocrit for 48 h in PBS or RPMI 1640 alone or RPMI 1640 with 50 mg/liter hypoxanthine or RPMI with 50 mg/liter hypoxanthine and 0.5% AlbuMAX II lipid-rich BSA. Erythrocytes were saponin lysed and washed twice with PBS prior to dsDNA quantification using a Quant-it PicoGreen dsDNA assay kit as described in Materials and Methods. Nondetectable values (below the limit of detection) were imputed as 0. Data in the left panel demonstrate that DNA abundance is concentration dependent and does not represent mere instrument noise. Data in the right panel demonstrate that components of media (such as AlbuMAX II lipid-rich BSA) contribute to DNA quantification but that erythrocytes in PBS contribute the majority of the measured DNA. Download FIG S1, TIF file, 1.7 MB. Copyright © 2018 Carey et al. 2018 Carey et al. This content is distributed under the terms of the Creative Commons Attribution 4.0 International license.

**FIG 3:**
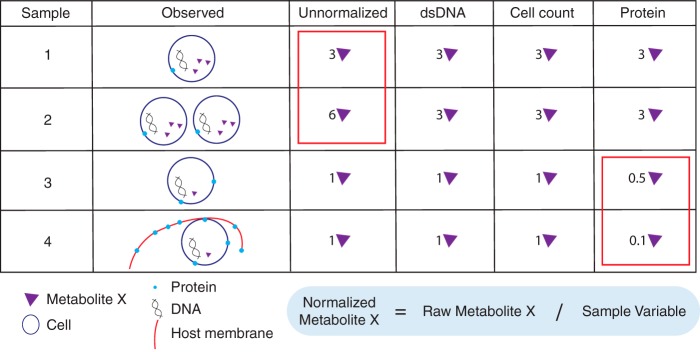
Normalization approaches impact the final metabolite abundance. Normalization controls for sample-to-sample variation were performed. Normalization requires sample metabolite abundance to be divided by the quantified normalization factor, the sample variable (the equation is in the blue box; normalization factors are shown left of the box). The examples of results shown in the table indicate abundances of X metabolites given several different sample metrics for normalization. For example, identical samples with different cell counts (sample 1 and sample 2) reveal the importance of normalization; without it, the data corresponding to the identical samples show a 2-fold difference in the values determined for metabolite X. The values determined for identical parasite samples 3 and 4 also show a nearly 2-fold difference in metabolite abundance after normalizing to protein levels, due to host bias for protein measures.

We normalized metabolomes with respect to these parasite-derived and host-derived metrics to determine if normalization reduces extraparasite noise to reveal parasite metabolomes. Normalization of metabolite levels can be calculated by a variety of methods (Table 1; Fig. 3), all aiming to enhance interpretation of results by controlling for technical or nonbiological variation. To normalize, we divide the value representing the abundance of each metabolite in a sample by the corresponding sample variable to control for sample-to-sample variation (Fig. 3). As illustrated in Fig. 3, normalization can significantly affect interpretation of results and should be selected carefully based on experimental design and knowledge of samples.

**TABLE 1:** Parameters in metabolomics analysis of intracellular parasites, including *Plasmodium*^a^

Parameter	Option(s)	Factor(s) to consider
Growth conditions	**Ring stage**	**Limited biomass (1–2 µm; Fig. 1A and B), haploid genome; few** **enrichment options**
	Late stage	Larger in size (3–10 µm), polyploid genome; can use magnetic enrichment (Fig. 1A)
	Mixed stages	Effects of stage variation on data
	Media batches	Relevant if using serum-based media formulations
	**Blood batches**	**Must be recorded and matched within comparisons (Table S3); useful** **to assess host contamination levels (Fig. 5 and 6)**
		
Additional controls	Uninfected erythrocytes	Used to identify or control for host metabolites; used in addition to normalization
		
Enrichment methods	**Saponin, other lytic reagents**	**Compatible with all stages (Fig. 1A); parasites remain in erythrocyte** **ghosts (Fig. 2A) (need improved methods that isolate parasite** **from host cell)**
	Magnetic purification	Increases parasite-to-host ratio (Fig. 1A)
		
Metabolite detection	NMR	Limited metabolite detection but higher confidence
	**Mass spectrometry**	**Industry standard for broad detection**
	Radio labeling	Targeted approach with high confidence
	Single metabolite assays	High-confidence, targeted approach with low throughput
		
Preanalysis normalization	Cell number normalization	Can be combined with any postanalysis normalization but requires sample manipulation
		
Postanalysis normalization	**Parasite-derived parameters**	**That is, parasite number; selection requires knowledge of** **experimental design**
	**Parameters with mixed** **derivation (host, parasite)**	**That is, protein, DNA; can fail to remove undesired noise (Fig. 2 and 4)**
	Internal standards	Dependent on metabolomics facilities
		
Centering	Mean	Standard centering
	**Median**	**Less sensitive to outliers**
	Other	See reference 54 for a summary of alternative approaches
		
Scaling	**Within-group SD**	**Requires no additional samples**
	Z-scoring	Requires control samples (i.e., untreated or uninfected erythrocytes)
		
Statistical analysis	**Univariate**	**Requires multiple-comparison corrections**
	**Multivariate**	**Reveals group differences based on multiple variables**
	**Machine learning (e.g.,** **random forest)**	**Classification more stringent than with univariate tests but can** **identify nonlinear effects**

a Note that most parameters do not have strict recommendations, as they are dependent on experimental design. Bolded text indicates methods that were employed and/or evaluated during this study. NMR, nuclear magnetic resonance.

**FIG 4:**
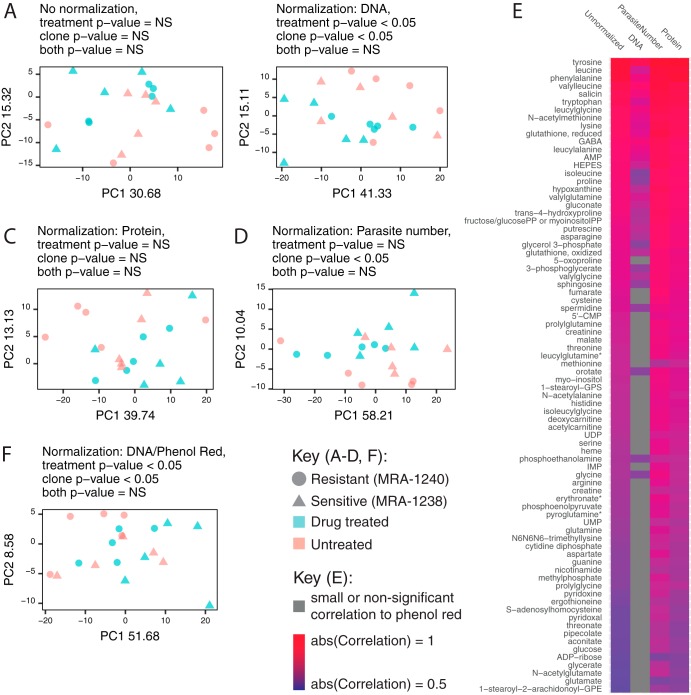
Metabolomes are dependent on the normalization approach and are influenced by extraparasite contamination. (A to D) Normalization affects metabolome similarity. (A to D) Principal-component (PC) analysis was performed prior to normalization (A) as well as after using three different normalization methods (DNA normalization [B], total protein normalization [C], and parasite count normalization [D]) on all identified metabolites. PERMANOVA significance is listed for each grouping. (E) Metabolites associated with components of media. The raw abundance of 82 metabolites was correlated with phenol red levels (unnormalized column), using a two-sided Pearson’s product moment correlation with Benjamini and Hochberg false-discovery rate correction. These associations were not removed with parasite number and protein normalization. DNA normalization best removes associations with components of media (increases in numbers of insignificant [gray] correlations); only 39% of correlations remain. (F) Removal of medium-associated metabolites. Principal-component analysis (PCA) of DNA-normalized samples with phenol red-correlated metabolites removed from the data set yielded no improvement in sample clustering.

Because the effect of normalization has not been explored in intracellular parasites, we normalized to parasite number (parasite derived), dsDNA amount (parasite, medium, and host derived), and total protein amount (parasite, medium, and host derived) and then performed principal-component analysis with all sample metabolomes (Fig. 4A to D). The normalization methods all yield distinct principal component structures, and yet none clearly separate the four sample groups (as measured by clustering of the sample groups by permutational multivariate analysis of variance [PERMANOVA]; *P* values are provided in the figure under the "Normalization" heading). However, with DNA normalization, we are able to separate drug-treated parasites from untreated parasites or clonal groups (Fig. 4B); with parasite number normalization, we can distinguish clonal groups (Fig. 4D).

Consistent with the lack of distinct separation, univariate statistical analysis revealed no metabolites that were differentially abundant among the four groups (see the supplemental material for code). When normalization is employed, metabolome differences between groups are highly dependent on the approach; the top differentially abundant metabolites are normalization method dependent (data not shown; see the supplemental material for code). These findings emphasize that biological interpretations can change significantly depending on the chosen analytic parameters and thus that the selected normalization metric is a critical parameter and must be shared for analytic reproducibility.

### Data filtering.

We next examined and removed extraparasite metabolites in our data set in order to explore the effect of sample contamination. Because there are no unique metabolites associated with the host, we explored medium-specific metabolites, specifically, phenol red and HEPES. Both phenol red (a pH indicator) and HEPES (a buffer) are components of the growth medium and should not be utilized by cells. These metabolites are routinely excluded from metabolomics analysis for this reason.

Interestingly, the abundances of 82 (of a total of 298) metabolites were correlated with phenol red (Fig. 4E) and the abundances of 76 metabolites were correlated with HEPES (data not shown); the abundances of 59 metabolites were correlated with both compounds. Many (>39%) of these metabolites remained correlated with the components of the media even after normalization (phenol red data are shown in Fig. 4E). Because phenol red and HEPES appeared to increase in abundance in drug-treated samples (nonsignificant trend; data not shown), we argue that this extraparasitic fraction may influence the interpretation of drug treatment data. If we remove these medium-associated metabolites from our analysis, surprisingly, sample separation into the four treatment groups does not provide an improvement in comparison to the results seen with DNA normalization alone (based on the remaining 216 metabolites; see Fig. 3F compared to Fig. 3B). Thus, both *post hoc* data filtering methods were insufficient to remove the effect of extraparasite contamination in our low-powered study.

### Machine learning.

**FIG 5:**
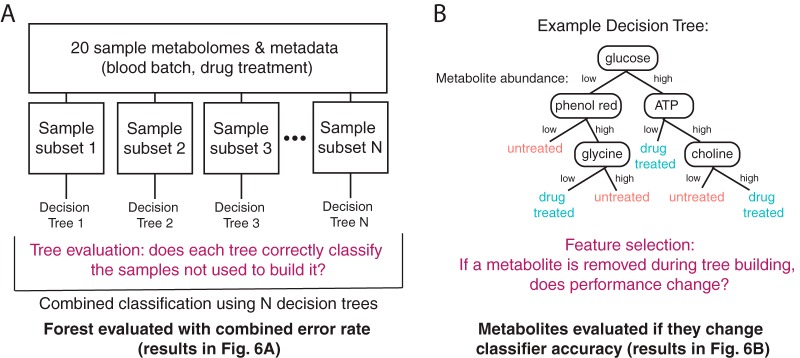
Random forest analysis. (A) Building a random forest classifier. Samples are randomly classified into subsets (training and test data sets); from the training subsets, decision trees are built to separate samples into groups (see panel B). Trees are evaluated by testing classification performance on the remaining samples from the test data sets. See Materials and Methods for more details on the analyses. (B) Evaluating metabolite importance. Metabolite importance is calculated by determining the effect of removal of the metabolite from the data set on classifier performance. See Materials and Methods for further details.

We next used machine learning to attempt to separate the extraparasite-associated metabolome from the parasite metabolome. Here, we leveraged the multiple blood batches used in parasite culture (Fig. 1A). Our four sample groups were grown in three different blood batches (Table S3). Univariate statistical analysis revealed only one metabolite with differential abundance results among the blood batches (1-arachidonoyl-GPE; see the supplemental material for code). To further explore the host contribution to the metabolome, we built random forest classifiers for analysis of blood batch and drug treatment data (Fig. 5). Random forest analysis is an internally validated machine learning approach, used here to classify samples into groups based on their metabolome (Fig. 5A) and to identify individual variables that are important for prediction accuracy (Fig. 5B).

**FIG 6:**
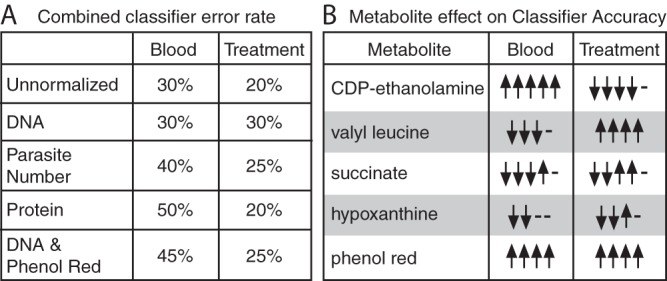
Blood batch and antimalarial treatment influence metabolomes. (A) Classifier performance. Classifiers were built to predict blood batch or treatment conditions using the metabolomics data with or without 4 normalization approaches. The classifier error rate varies with the normalization approach. (B) The normalization method determines the important metabolites. A sample consisting of five metabolites associated with improved or worsened classifier accuracy is shown. These metabolites are shown in accordance with their importance in classifier performance and their interesting behavior across classifiers. Upward-pointing arrows indicate that the metabolite improves classifier accuracy in one classifier, and downward-pointing arrows indicate they worsen accuracy in one classifier (arrows represent the normalization approaches from panel A); if the metabolite does not improve or worsen accuracy, a dash is shown. Contradictory results (both upward-pointing and downward-pointing arrows for one metabolite) indicate that the normalization method changes the importance of the metabolite. Note that valyl leucine, hypoxanthine, and phenol red were removed upon phenol red filtering and, therefore, are present in only 4 classifiers, as indicated by the four arrows and dashes.

We first built classifiers for analysis of blood batch data across all samples. Ninety-five metabolites (of 298) improved classifier accuracy in analysis of blood batch data (using the DNA normalized data set; see the supplemental material for code). Many of these metabolites are correlated in abundance with the components of the media explored (Fig. 4E), including CDP-ethanolamine, AMP, ADP-ribose, and aspartate, which are among the top 10 most influential metabolites in this classifier. The remaining metabolites (203 in total) had no effect on the performance of the classifier or worsened its predictive ability, indicating they are not associated with blood batch due to high variability or association with other features that differentiate samples. The classifier built from DNA-normalized metabolomes predicted blood batch data with a 30% error rate (Fig. 6A). We also built a blood batch classifier from each of the other normalization approaches (Fig. 6A).

To determine if blood batch is as influential on the metabolome data as a potent antimalarial drug treatment, we built similar classifiers for analysis of artemisinin treatment. Parasites were classified into two treatment conditions with a 30% class error rate using DNA-normalized metabolomes (Fig. 6A). A total of 118 metabolites (of 298) improved the accuracy of this classification, including medium-correlated metabolites such as pipecolate, several dipeptides, and phenol red (see the supplemental material for code).

The performance of our classifier (Fig. 6A) was relatively poor due to the small sample size, and the results indicated that only a subset of the measured metabolomes was predictive of blood batch or drug treatment. Classifiers built from data under alternative normalization approaches were comparable in performance, but different metabolites contributed to their accuracy (Fig. 6B). Removal of phenol red and associated metabolites from the data set (listed as phenol red correction data; Fig. 6A) reduced blood batch classifier performance more than it did treatment classifier performance; this result is not surprising, because both the components of the media and the host cells are extraparasitic. Thus, by removing medium contamination, we may also be removing host contamination and data associated with the blood batch. However, phenol red is associated with both blood batch classifier accuracy and treatment classifier accuracy (Fig. 6B); this result supports the idea of the necessity of removing extraparasitic metabolites during sample preparation, as they can skew meaningful biological interpretation.

Interestingly, when the classifier was built using a different normalization approach, the set of metabolites that most extensively contributed to accuracy changed (representative examples are shown in Fig. 6B; code for the full analysis is available in the supplemental material). Although some metabolites (such as CDP-ethanolamine or valyl leucine) were consistently associated with blood classifier accuracy or treatment classifier accuracy, respectively, some metabolites (such as succinate and hypoxanthine) gave contradictory results depending on the data normalization approach (Fig. 6B). 1-Arachidonoyl-GPE, identified by univariate statistics, was not among the top most predictive metabolites in any classifier but did contribute to accuracy in some blood batch classifiers. Thus, sample metabolome can classify both blood batch and sample group, indicating that sample treatment and blood batch influence the metabolome and that this is normalization approach dependent.

## DISCUSSION

The lifestyle of intracellular parasites presents challenges to implementing traditional metabolomics protocols, predominately due to host metabolite contamination and limitations in the amounts of parasite material. These challenges are exacerbated when studying early parasite stages (such as the *Plasmodium* ring stage studied here), when the parasite is smallest. In our study, we conducted a detailed assessment of the impact of extraparasite contamination and investigated analytic approaches to improve metabolome interpretation. We recommend improved discussion of normalization methods in the metabolomics field, especially for intracellular parasites, as normalization significantly effects the interpretation of a data set. Additionally, we propose several analytic approaches to explore the effect of host contamination.

### Metabolome interpretation is normalization approach dependent.

Normalization limits nonbiological variation and is absolutely essential for biological interpretation (Fig. 3). Normalization factors can be calculated using a variety of methods, and normalization is implemented either before or after metabolite quantification and identification (described as preanalysis or postanalysis) (Table 1) (21, 22). Often, preanalysis normalization is conducted by isolating the same number of cells for analysis (23) but this is not typically used in the study of P. falciparum as generating adequate biomass can be challenging (3, 5, 12). Furthermore, the use of inaccurate quantification methods may negate the utility of this step by introducing more variability. Postanalysis normalization methods are also routinely used; these include the use of internal standards (4, 21), corrections for protein amounts (often used for supernatant or cell-free metabolomics [24]), DNA content (an approach validated in mammalian cells [25] and applied to bacterial cells [26]), or cell number (typically used for bacterial populations [27]).

To our knowledge, normalization was never described in detail in previous metabolomics studies of P. falciparum, perhaps due to the technical challenges that we explored here. We evaluated three postanalysis normalization approaches, namely, the protein, double-stranded DNA, and parasite number approaches (Fig. 3 and 4A to D). Overall, we conclude that normalization significantly affects the interpretation of results (Fig. 4 and 6). The normalization approach influences the metabolites with the greatest differential abundances (data not shown because they did not reach significance) and the metabolites predictive of sample group shift with data normalization (Fig. 6).

In the present studies, only the parasite count data were entirely parasite derived. The extracellular environment (including components of media and host erythrocyte) likely contributes heavily to protein abundance. Accordingly, parasite count and protein abundance are not correlated. We also show that the host cell contributes to dsDNA levels, despite lacking a nucleus (see Fig. S1 in the supplemental material). This material may be contributed by the small proportion of dying white blood cells that remain after erythrocyte preparation. Despite this finding, our analysis shows that dsDNA normalization of early-ring-stage metabolomes best distinguished sample and treatment groups and removed medium contamination (Fig. 4). Much variability still remained after this step; we did not identify any differentially abundant metabolites even though artemisinin has been reported to have metabolic effects on late-stage parasites (3, 5, 16) and dormancy induces metabolic shifts in ring-stage parasites (17–19). Although dsDNA normalization was the most effective approach in our data set, it is not appropriate for all experimental cases; for example, this type of analysis would introduce variability in comparisons of groups of different parasite stages due to known genome copy number differences (28, 55).

### Media and host contribute to the measured metabolome.

We found that extraparasite material contributed by host erythrocytes and components of media can also heavily impact the metabolome. Many studies employ erythrocyte lysis prior to sample purification (3, 8, 9, 12) (see Materials and Methods). However, several results from our study show that this step does not eliminate the potential for host contamination.

First, lipid species were the major class of metabolites detected in our analysis (Fig. 1C), perhaps due to the abundance of the erythrocyte membranes or “ghosts” present in the preparations (Fig. 2A). Second, more than a quarter of the metabolome is correlated with the components of the media (phenol red [Fig. 4E] and HEPES [data not shown]). Unlike HEPES (11), phenol red has not been shown to be imported into the parasite; neither metabolite is produced or biochemically consumed by the parasite. Thus, it is likely that these medium-derived metabolites remained associated with cells following *in vitro* culture in medium. This medium also contains high levels of other metabolites such as glutathione, hypoxanthine, glutamine, and many amino acids, which are correlated with phenol red and/or HEPES abundances. Third, we measured metabolites not expected to be produced or consumed by *Plasmodium* (2). For example, kynurenine is present in erythrocytes, derived from the amino acid l-tryptophan (29, 30), and is not known to be involved in P. falciparum metabolism (31). Lastly, the only differentially abundant metabolite in our entire analysis that reached significance was associated with the blood batch (1-arachidonoyl-GPE). This metabolite has not been studied in the context of erythrocyte or *Plasmodium* metabolism but can be explored as a potential marker of host contamination.

In fact, we were able to predict a set of metabolites that are most likely to be influenced or derived from the host erythrocyte by identifying the metabolites that are most predictive of blood batch (Fig. 5B and 6B; see figures in the supplemental material code for a comprehensive list). Going forward, it may be possible to use specific metabolite markers to assess levels of host contamination and parasite sample purity and to control for host contamination during analysis.

### Future recommendations.

Parasite metabolomics is a rapidly expanding field; thus, well-documented methodologies and rigorous evaluation criteria will enhance data reproducibility and the quality of metabolomics-derived observations. In this study, we compiled evidence of host erythrocyte and medium contamination in untargeted metabolomics studies of intracellular parasites and explored the analytical decisions that influence metabolome interpretation. We showed that analytic approaches can improve the accuracy and interpretability of intracellular parasite metabolomes but that, ultimately, better methods are needed to extract biological differences from samples.

A common approach used in the study of P. falciparum involves the use of an uninfected erythrocyte control to adjust for the presence of host metabolites (4, 6, 7, 9–11), but even with the use of this control, interpretation of data remains challenging (see, e.g., reference 32). Uninfected erythrocyte controls are used for *z* score metabolite abundance calculations (infected relative to uninfected), for differential abundance calculations (infected divided by uninfected), or for calculations involving subtraction of “host” metabolite data from infected-population data. However, we hypothesize that, in some cases, the use of the uninfected erythrocyte control alone is not sufficient; as we show in Fig. 4F and 6, correcting the data set by removing extraparasite contamination data (medium-associated metabolites) fails to improve treatment classification. We suggest that the quantitative analytic methods applied here must also be used to evaluate the efficacy of the uninfected erythrocyte control.

Another common analytic step involves the removal of extraparasitic metabolites, such as phenol red, as they are considered to represent noise from culture media. However, these metabolites contain valuable information about experimental variation and could be used for quality control, as indicated by the frequent correlation between phenol red abundance and other metabolites (Fig. 4E). For this reason, these metabolites should not be excluded from the data set and subsequent analysis.

We suggest a set of considerations and recommendations for enhancing the accuracy of parasite metabolomics (Table 1 and below). First, samples must be better purified away from host material. Purification could involve enrichment methods to increase parasitemia prior to lysis (reducing the ratio of uninfected host cells to parasites) or the direct removal of host material postlysis. Currently, enrichment approaches exist only for late-stage malaria parasites. Second, markers of host contamination must be used to evaluate the level of medium and host contamination. The number of metabolites with abundances correlated with phenol red or HEPES can be used to assess the contribution of the media. The visual detection of ghost material (via microscopy) combined with assessment of host-specific metabolite markers is an effective option to assess sample purity. Additionally, analytic approaches (such as blood batch classification) can be used to identify remaining or experiment-specific markers of contamination. Finally, data must be normalized to appropriate measurements to maximize the metabolome signal associated with the treatment of interest; subsequent subtraction of metabolites associated with host or media (e.g., uninfected erythrocyte control or known components of media) can further reduce metabolite influence mediated by extraparasite conditions. Importantly, we propose that, similarly to studies in *Leishmania* (33–35), normalization and discussion of the chosen normalization metrics should become standard during metabolomics analysis of intraerythrocytic parasites. With these considerations, metabolomics has the potential to become a powerful tool in the study of intracellular parasites.

## MATERIALS AND METHODS

### Parasite cultivation.

Laboratory-adapted P. falciparum clonal lines were cultured in RPMI 1640 (Thermo Fisher Scientific, Waltham, MA) containing HEPES (Sigma-Aldrich, St. Louis, MO) supplemented with 0.5% AlbuMAX II lipid-rich bovine serum albumin (BSA) (Sigma-Aldrich, St. Louis, MO) and 50 mg/liter hypoxanthine (Thermo Fisher Scientific, Waltham, MA). Parasite cultures were maintained at 3% hematocrit and diluted with human red blood cells (blood batch noted in Table S3) to maintain parasitemia at between 1% and 3%, with changes of culture medium every other day (Fig. 1A; step 1). Cultures were incubated at 37°C with 5% oxygen, 5% carbon dioxide, and 90% nitrogen (36). Some samples were treated with artemisinin, an antimalarial with metabolic effects (dihydroartemisinin; see antimalarial treatment details in Table S3) (3, 5). Cultures were tested for mycoplasma monthly using a LookOut Mycoplasma PCR detection kit (Sigma-Aldrich); none tested positive.

### Parasite isolation.

Two distinct laboratory-adapted clinical isolates of P. falciparum (BEI Resources, NIAID, NIH; Plasmodium falciparum strains IPC 5202/MRA-1240 and IPC 4884/MRA-1238, contributed by Didier Ménard) containing mixed stages with >50% rings were synchronized using 5% sorbitol (Sigma-Aldrich, St. Louis, MO) (37). The resultant early-stage cultures were incubated at 37°C in AlbuMAX media to allow the development of a predominantly schizont population. After the late-stage population was confirmed using microscopy, cultures were checked every 1 to 2 h for the development of newly invaded ring-stage parasites. If the parasites were treated with dihydroartemisinin, the treatment was performed at this stage. Fourteen 25-cm^3^ flasks containing early-ring-stage parasites (<3 h postinvasion, treated with dihydroartemisinin or left untreated) were subsequently lysed from the erythrocyte membrane using 0.15% saponin, as previously described (38) (Fig. 1A; step 3). Prior to lysis, a sampling of parasite material was taken for determination of erythrocyte count (hemocytometer) and parasitemia (Sybr green-based flow cytometry [39]), which contributed to parasite number determination (total number of erythrocytes × percent parasitemia yields the total parasite count). Additional samples were obtained following erythrocyte lysis for protein quantification using Bradford reagent (Sigma-Aldrich, St. Louis, MO). A series of three wash steps were then performed using 1× phosphate-buffered saline (PBS) (Sigma-Aldrich, St. Louis, MO) and centrifugation at 2,000 × *g* to remove soluble erythrocyte metabolites. Purified material was kept on ice until it was flash frozen using liquid nitrogen (to quench metabolism), followed by storage at −80°C until sent for analysis. This procedure was performed five times for both parasite clonal lines (strains IPC 5202/MRA-1240 and IPC 4884/MRA-1238) to provide 10 drug-treated replicates for metabolomic analysis. Additionally, matched parasites (same parasite lineage, medium type, stage, blood batches, and purification methods) were also grown without drug treatment (Table S3) to generate 10 additional control samples (see comparison in Fig. 1B).

### Metabolite preparation, analysis, and identification.

Metabolites were identified using ultra-high-performance liquid chromatography coupled with tandem mass spectroscopy (UPLC/MS-MS) by Metabolon, Inc. (Durham, NC). All sample preparations and metabolite identifications were performed according to standard protocols of Metabolon, Inc. (briefly summarized here). Double-stranded DNA was quantified in all samples using a Quant-it PicoGreen dsDNA assay kit (Thermo Fisher, Waltham, MA) according to the manufacturer’s instructions. Proteins were precipitated using methanol for 2 min with vigorous shaking and then centrifuged for extraction (Fig. 1A; step 4). Sample extracts were separated into aliquots, dried, and suspended in appropriate standard-containing solvents for analysis by four methods. These four methods facilitate the measurement of metabolites with different biochemical properties and include two reverse-phase UPLC/MS-MS methods, one with positive ion electrospray ionization (ESI) optimized for hydrophilic compounds and one optimized for hydrophobic compounds, and a third method with negative-ion-mode ESI. Additionally, a UPLC/MS-MS method with negative-ion-mode ESI following elution from a hydrophilic interaction chromatography column was used. Waters Acquity ultraperformance liquid chromatography and a Thermo Scientific Q Exactive high-resolution/accurate mass spectrometer were used for all metabolite detection procedures (Fig. 1A; step 5).

To evaluate the quality of the mass spectrometry pipeline, several controls were used. Ultrapure water or the solvent alone or both were used as blank samples to control for nonspecific signals in the pipeline. Technical controls were employed to ensure that the instruments were working within specifications; a pooled sample of human plasma and a pooled aliquot of experimental samples were used to distinguish biological from technical variability. A set of recovery and internal standards were also used to quantify variability and instrument performance. Variability scores for all runs included in this analysis met the acceptance criteria specified by Metabolon, Inc.

Raw data were extracted using hardware and software developed by Metabolon, Inc. Metabolites were quantified using the area under the curve and were identified by comparison to a library of several thousands of preexisting entries of purified standards or recurrent unknown compounds. Each library standard was uniquely authenticated by retention time/indices, mass-to-charge ratios, and chromatographic data. Named metabolites corresponded to library standards or were predicted with confidence according to the standard protocols specified by Metabolon, Inc.

### DNA quantification.

Measurement of host-derived dsDNA levels was performed by incubating uninfected erythrocytes at 3% hematocrit for 48 h in PBS or RPMI 1640 alone or RPMI 1640 with 50 mg/liter hypoxanthine or RPMI with 50 mg/liter hypoxanthine and 0.5% AlbuMAX II lipid-rich BSA. Erythrocytes were subjected to saponin lysing and washed prior to dsDNA quantification using a Quant-it PicoGreen dsDNA assay kit as described above.

### Microscopy.

Laboratory-adapted P. falciparum clones (BEI Resources, NIAID, NIH; Plasmodium falciparum, patient line strain E/MRA-1000 or strain IPC 5202/MRA-1238, contributed by Didier Ménard) at 1.5% parasitemia with >60% rings were lysed using 0.15% saponin, as previously described (38). Samples were washed twice using 1× PBS (Sigma-Aldrich, St. Louis, MO) and centrifugation at 2,000 × *g* for 5 min. For bright-field images, parasites were fixed with methanol and stained with Giemsa stain for 15 min. Images were obtained on a Nikon Eclipse Ci microscope (×100) using an Imaging Source microscope camera and Nikon NIS Elements imaging software. Representative images are shown. For production of fluorescent images, samples were stained on slides with either DAPI (4′,6-diamidino-2-phenylindole) (Sigma-Aldrich, St. Louis, MO) at 1:20,000 or CD235a-phycoerythrin (CD235a-PE) antibody (Thermo Fisher Scientific, Waltham, MA) at 1:100. Fluorescent images were acquired using an Evos FL cell imaging system (Thermo Fisher Scientific, Waltham, MA). Representative images are shown, and quantification of 1,214 parasites associated with erythrocyte membranes was performed for 11 preparations.

### Data preprocessing and statistical analysis.

Following the analytical protocol outlined in reference 40, we first preprocessed metabolite abundances for each sample by imputing missing values corresponding to half of the lowest detectable metabolite abundance. Next, we normalized metabolite abundances by sample features (Fig. 3), followed by normalization using metabolite features with log transformation, centering, and scaling (Fig. 1A, step 6) (41).

Specifically, to limit intersample variability, metabolite abundances for each replicate were normalized to the sample value for double-stranded DNA, protein, or parasite number. To limit intermetabolite variability, metabolite abundances were log transformed, centered with respect to the median (42), and scaled by standard deviation (Fig. 1A; step 6).

The resultant processed metabolite abundances were used for calculation of univariate and multivariate statistics, as well as for classification. All analyses were conducted using R with tidyverse (43), knitr (44), reshape2 (45), pracma (46), grid and gridExtra (47), extrafont (48), and RSvgDevice (49) for data wrangling and visualization and vegan (50) and base R (51) for analysis. Analyses of variance (ANOVAs) were used to compare group means for determinations of differential abundances, and *P* values were adjusted using the false-discovery rate (Benjamini and Hochberg) (52) to correct for multiple testing. The significance cutoff was 0.05. PERMANOVAs were used to compare population separation data (Fig. 4A to D and F). Correlations were conducted using a two-sided Pearson’s product moment correlation with false-discovery rate (Benjamini and Hochberg) in R. See the supplemental material for code documenting a detailed analysis.

### Random forest analysis.

Random forest analysis is a machine learning technique and was used here to classify sample groups (Fig. 5A). Within a random forest classifier, individual trees are built from subsets of the data and internally validated with respect to the remaining data set (Fig. 5A). With this approach, variables (metabolites) are ranked by their effect on classifier accuracy, as measured by a change in performance following removal of the variable (Fig. 5B). Classifiers were built with each data normalization method to predict drug treatment or blood batch. These analyses were conducted in R using the RandomForest package and base R (51, 53). See the supplemental material for code and detailed analysis.
