# Supplementary material for: Influential Parameters for the Analysis of Intracellular Parasite Metabolomics
Source: mSphere. 2018 Apr 18;3(2):e00097-18. doi: 10.1128/mSphere.00097-18 (PMC5907652; doi:10.1128/mSphere.00097-18)
Supplement: TABLE S2 [file sph002182519st2.docx]

| Image preparation date | Free Parasites | Total Parasites Counted |
| --- | --- | --- |
| 8.22.17 | 3 | 21 |
| 9.22.17 | 34 | 77 |
| 9.25.17 | 20 | 58 |
| 9.25.17 | 36 | 78 |
| 9.27.17 | 10 | 49 |
| 9.27.17 | 23 | 58 |
| 12.15.17 | 41 | 242 |
| 12.16.17 | 37 | 153 |
| 1.9.18 | 60 | 131 |
| 1.11.18 | 65 | 204 |
| 1.12.18 | 50 | 143 |
| TOTAL | 379 | 1214 |
