# Supplementary material for: Influential Parameters for the Analysis of Intracellular Parasite Metabolomics
Source: mSphere. 2018 Apr 18;3(2):e00097-18. doi: 10.1128/mSphere.00097-18 (PMC5907652; doi:10.1128/mSphere.00097-18)
Supplement: TABLE S3 [file sph002182519st3.docx]

| Sample ID | Clone | Blood batch | Antimalarial treatment | Protein (mg) | DNA amount (mg/ml) | Parasitemia (%) | Parasite number | Stage distribution |
| --- | --- | --- | --- | --- | --- | --- | --- | --- |
| BAT-A (+) | MRA-1240 | 1 | 700nM DHA | 91.0 | 0.927 | 1.1 | 3.27E+06 | 97% early rings |
| BAT-A (-) | MRA-1240 | 1 | None | 67.1 | 0.587 | 1.1 | 3.27E+06 | 97% early rings |
| BAT-B (+) | MRA-1240 | 1 | 700nM DHA | 121.2 | 0.476 | 1.0 | 2.89E+06 | 98% early rings |
| BAT-B (-) | MRA-1240 | 1 | None | 118.1 | 1.216 | 1.0 | 2.89E+06 | 98% early rings |
| BAT-C (+) | MRA-1240 | 2 | 700nM DHA | 119.2 | 0.985 | 1.0 | 2.93E+06 | 98% early rings |
| BAT-C (-) | MRA-1240 | 2 | None | 87.9 | 1.739 | 1.0 | 2.93E+06 | 98% early rings |
| BAT-D (+) | MRA-1240 | 2 | 700nM DHA | 98.3 | 0.656 | 1.9 | 6.95E+06 | 98% early rings |
| BAT-D (-) | MRA-1240 | 2 | None | 130.1 | 0.557 | 1.9 | 6.95E+06 | 98% early rings |
| BAT-E (+) | MRA-1240 | 3 | 700nM DHA | 125.9 | 1.326 | 2.2 | 6.51E+06 | 93% early rings |
| BAT-E (-) | MRA-1240 | 3 | None | 128.5 | 2.083 | 2.2 | 6.51E+06 | 93% early rings |
| PUR-A (+) | MRA-1238 | 1 | 700nM DHA | 120.2 | 0.325 | 0.6 | 1.31E+06 | 96% early rings |
| PUR-A (-) | MRA-1238 | 1 | None | 125.4 | 0.547 | 0.6 | 1.31E+06 | 96% early rings |
| PUR-B (+) | MRA-1238 | 1 | 700nM DHA | 123.3 | 0.259 | 1.0 | 2.50E+06 | 98% early rings |
| PUR-B (-) | MRA-1238 | 1 | None | 121.0 | 0.673 | 1.0 | 2.50E+06 | 98% early rings |
| PUR-C (+) | MRA-1238 | 2 | 700nM DHA | 104.6 | 0.648 | 0.6 | 2.26E+06 | 97% early rings |
| PUR-C (-) | MRA-1238 | 2 | None | 100.4 | 0.543 | 0.6 | 2.26E+06 | 97% early rings |
| PUR-D (+) | MRA-1238 | 3 | 700nM DHA | 120.7 | 0.599 | 1.0 | 3.32E+06 | 96% early rings |
