# Supplementary figures and images for: Influential Parameters for the Analysis of Intracellular Parasite Metabolomics
Source: mSphere. 2018 Apr 18;3(2):e00097-18. doi: 10.1128/mSphere.00097-18 (PMC5907652; doi:10.1128/mSphere.00097-18)

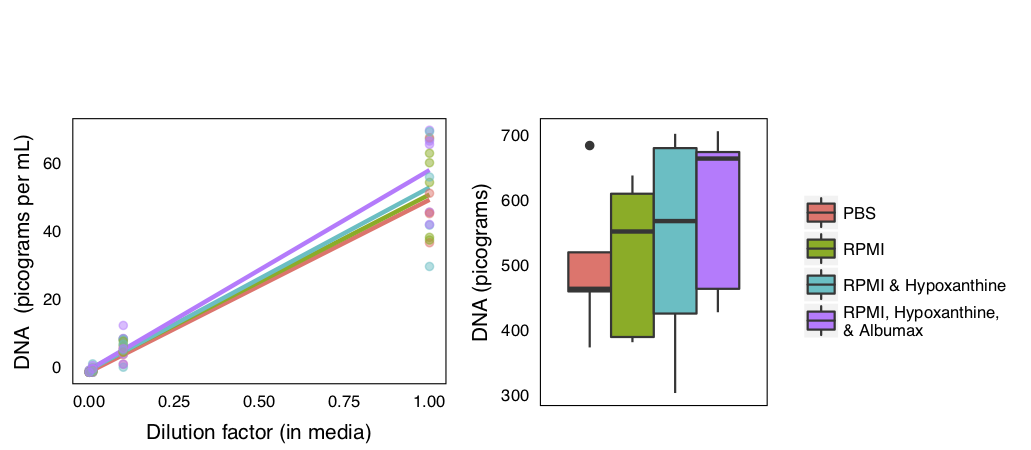

Supplement: FIG S1 [file sph002182519sf1.tif]
